# Lyme Carditis: A Reversible Culprit in Non-Ischemic Cardiomyopathy

**DOI:** 10.7759/cureus.78770

**Published:** 2025-02-09

**Authors:** Krunal Shukla, Eric J Basile, Jonathan Van Name, Nisarg Ray

**Affiliations:** 1 Internal Medicine, University of Florida College of Medicine, Gainesville, USA

**Keywords:** cardiomyopathy, heart failure, lyme carditis, non ischemic cardiomyopathy, tick-borne infections

## Abstract

Lyme disease (LD), caused by *Borrelia burgdorferi*, is a tick-borne illness that can lead to Lyme carditis, which most commonly presents as a high-degree atrioventricular (AV) block. While conduction abnormalities are well-documented, LD has also been implicated in non-ischemic cardiomyopathy, though this manifestation remains rare and under-recognized. We present the case of a 57-year-old female with newly diagnosed heart failure with reduced ejection fraction (HFrEF) and first-degree AV block, who initially presented with nausea, dizziness, fatigue, and gastrointestinal symptoms. Her history included multiple tick bites, subacute joint pain, and intermittent nonspecific rashes. Initial transthoracic echocardiography (TTE) demonstrated severe global hypokinesis with a left ventricular ejection fraction (LVEF) of 10-15%. Extensive ischemic and inflammatory workups, including coronary angiography and cardiac MRI, were unremarkable. However, Lyme serology was positive, and the patient was started on a 21-day course of doxycycline alongside guideline-directed heart failure therapy. A follow-up TTE months later demonstrated remarkable recovery, with LVEF improving to 55-59% and resolution of wall motion abnormalities. This case highlights the importance of considering LD as a reversible cause of non-ischemic cardiomyopathy, particularly in patients with risk factors such as tick exposure and relevant clinical symptoms. Early recognition and appropriate antimicrobial treatment can lead to significant cardiac recovery, underscoring the need for a high index of suspicion when evaluating new-onset systolic dysfunction with an otherwise negative ischemic workup.

## Introduction

Lyme disease (LD) is a tick-borne illness caused by a species of the *Borrelia* genus, most commonly *Borrelia burgdorferi*. If left untreated, LD progresses through three phases: early localized, early disseminated, and late disseminated. Cardiovascular complications are most common during the early disseminated and late disseminated phases, typically presenting as high-degree atrioventricular (AV) block. Lyme carditis is seen in 1.5%-10% of all patients with Lyme borreliosis in the United States, with the majority developing cardiac conduction abnormalities. Another potential long-term consequence of untreated or poorly managed LD is dilated cardiomyopathy [1,2]. Lyme-associated cardiac manifestations have largely been attributed to immune-mediated injury due to possible cross-reaction between *Borrelia burgdorferi* antigens and cardiac epitopes.

The diagnostic workup for Lyme carditis includes a 12-lead electrocardiogram (ECG), with or without 24-hour cardiac telemetry, to detect PR interval prolongation. Echocardiography is used to identify wall motion abnormalities. Serologic testing follows a two-tiered approach, beginning with enzyme-linked immunosorbent assay (ELISA) and confirmation through Western immunoblot testing. It is important to note that ELISA results may be negative in both immunocompetent and immunocompromised patients during the early phases of LD. The false negative rate for Lyme disease ELISA testing is high, up to 50% in the early stage (0-4 weeks), because antibodies may not have developed yet. In later stages, the false negative rate drops to around 10-20%. False negatives occur due to early testing, immune suppression, strain variability, and technical limitations. To improve accuracy, the Centers for Disease Control and Prevention (CDC) recommends confirming ELISA results with a Western blot. A positive IgM Western immunoblot requires the presence of at least two of three bands [2,3]. Test results must be interpreted in conjunction with the patient’s history and clinical presentation due to variability in serologic and immunoassay findings. The optimal treatment for LD involves eliminating the infectious pathogen using antimicrobial agents, with doxycycline or ceftriaxone being the preferred choice depending on the clinical phase of the disease [2]. 

In this report, we present a rare case of dilated cardiomyopathy with overt left-sided heart failure symptoms and first-degree AV block in an immunocompetent patient. Remarkably, the patient experienced recovery of left ventricular ejection fraction following treatment for LD. This case underscores the importance of considering tick-borne diseases, particularly LD, as a potential cause of dilated cardiomyopathy, especially in patients with relevant clinical history and presentation. 

## Case presentation

A 57-year-old female with a history of newly diagnosed heart failure with reduced ejection fraction (HFrEF) and obstructive sleep apnea (OSA) presented to the emergency department with nausea, dizziness, right lower quadrant (RLQ) pain, constipation, and fatigue. Of note, she was a Florida resident without any recent travel history. Her initial presentation of acute heart failure was preceded by multiple tick bites over the course of the past two months. The patient also reported a subacute history of widespread joint pain along with intermittent red rashes, but no specific bullseye rash. Her hospital admission was also complicated by a urinary tract infection (UTI). A tick-borne infectious panel was ordered out of concern for tick-borne illness.

Initial laboratory studies revealed a positive Lyme IgM antibody (41 kDa, 23 kDa, and 39 kDa), while Lyme IgG and total Lyme antibody were unremarkable. Additional testing for *Anaplasma*, *Ehrlichia*, *Babesia*, and Rocky Mountain spotted fever (RMSF) antibodies were negative. Routine laboratory studies, including a complete blood count, basic metabolic panel, and lipase, were significant only for mild non-oliguric acute kidney injury (Table 1). The kidney injury was initially believed to be secondary to dehydration, but upon further cardiac work-up and clinical concern for hypervolemia, it was most consistent with cardiorenal syndrome, which resolved with low-dose intravenous diuresis using furosemide 20 mg.

**Table 1:** Initial admission laboratory test results and infectious workup. WBC: White blood cells

Laboratory test results	Values	Units	Reference Range
Sodium	138	mmol/L	135-145 mmol/L
Potassium	3.8	mmol/L	3.5-5.1 mmol/L
Urea nitrogen	46	mg/dL	6-20 mg/dL
Creatinine	1.68	mg/dL	0.6-1.2 mg/dL
Lipase	91	U/L	0-160 U/L
B. burgdorferi Ab, total	0.55	-	< 0.90 (Negative)
Rocky mountain spotted fever IgG	<1:64	-	< 1:64 (Negative)
E. Chaffeensis IgG	<1:64	-	< 1:64 (Negative)
Babesia Ab IgG	<1:16	-	< 1:64 (Negative)
Lyme IgG Line Blot	Negative	-	Negative
Lyme IgM Line Blot	Positive (41, 39, 23 kDa)	-	Negative
WBC	6.5	10^3^/uL	4.0-11.0 x 10^3^/uL
Hemoglobin	14.3	G/dL	13.5-17.5 g/dL
Platelet	226	10^3^/uL	150-400 x 10^3^/uL

**Figure 1:**
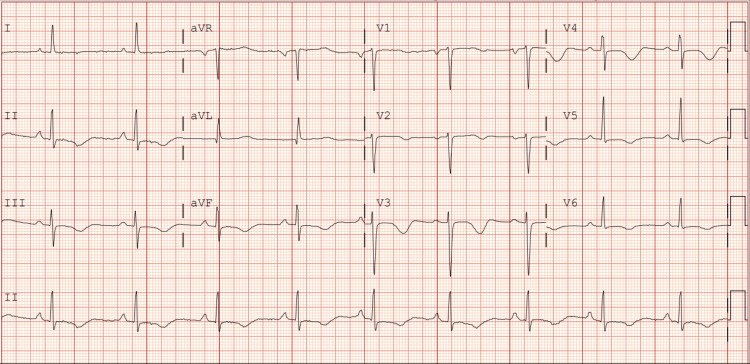
Admission EKG demonstrating a prolonged PR interval consistent with first-degree atrioventricular block. EKG: Electrocardiogram

**Figure 2:**
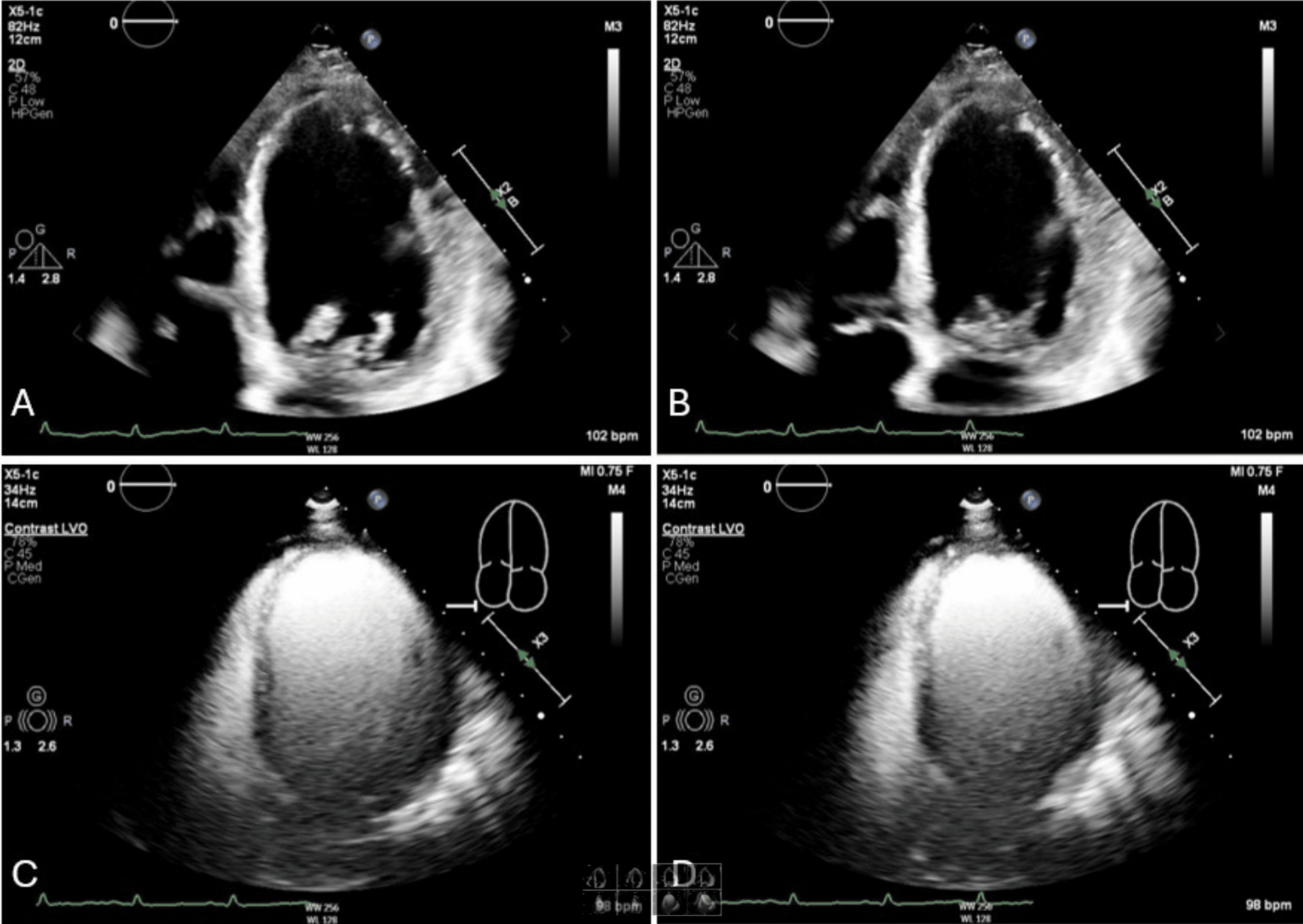
Echocardiogram images of the apical four-chamber view during (A) diastole and (B) systole, as well as left ventricular opacification views during (C) diastole and (D) systole, demonstrate a severely reduced left ventricular ejection fraction (10–15%) prior to doxycycline treatment.

**Figure 3:**
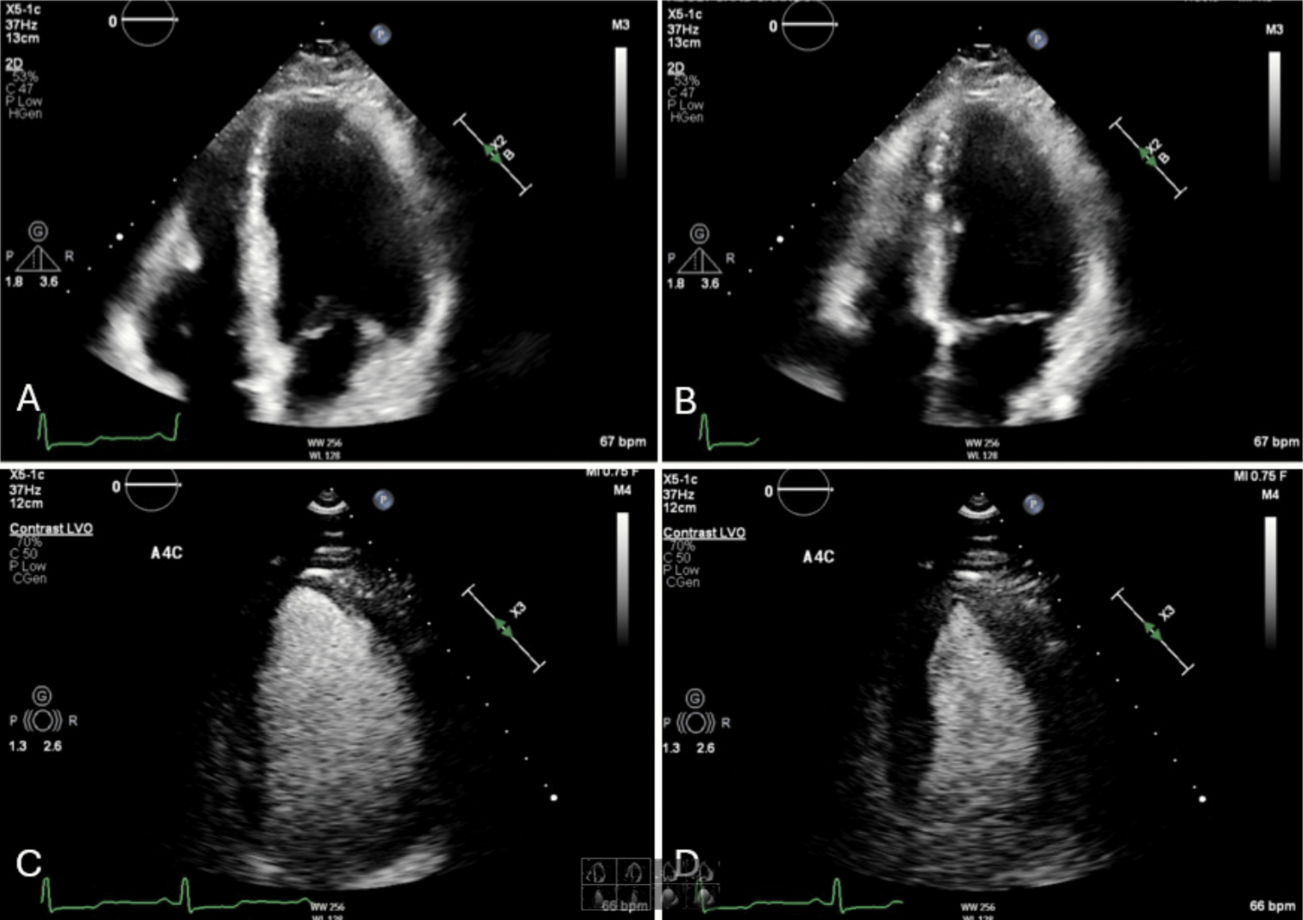
Echocardiogram images of the apical four-chamber view during (A) diastole and (B) systole, as well as left ventricular opacification views during (C) diastole and (D) systole, demonstrate a significant recovery in left ventricular ejection fraction (50–55%) with no observed wall motion abnormalities following treatment with doxycycline.

Electrocardiography at the time of admission demonstrated a prolonged PR interval of 210 milliseconds consistent with first-degree atrioventricular (AV) block (Figure 1). The initial transthoracic echocardiogram (TTE) showed a severely reduced left ventricular ejection fraction (10-15%) with severe global hypokinesis and moderate left ventricular dilation (Figure 2). A myocardial perfusion test did not show any regions concerning ischemia. Additionally, cardiac catheterization showed no evidence of coronary artery disease (CAD). A cardiac MRI with gadolinium enhancement was ordered to evaluate for myocarditis which resulted in an unremarkable study. Given the positive Lyme serologies and constellation of symptoms, the patient was started on doxycycline 100 mg two times a day for 21 days. The patient completed a full course of doxycycline and was started on guideline-directed medical therapy in the form of losartan, metoprolol succinate, and empagliflozin. A repeat TTE four months later demonstrated significant improvement in ejection fraction to 55-59% with no wall motion abnormalities (Figure 3). With no obstructive coronary disease, a significant history of tick bites, and positive Lyme IgM, it was concluded that her non-ischemic cardiomyopathy is most likely attributable to Lyme disease. 

## Discussion

Lyme carditis is a rare but reversible cause of acute-onset congestive heart failure. Lyme carditis-associated heart failure has been reported in only a few cases, with most presentations involving conduction abnormalities. Although the exact mechanism through which Lyme disease causes cardiac pathologies is not well understood, there is believed to be at least some component of immune-mediated injury from cross-reaction between *Borrelia burgdorferi* antigens and cardiac epitopes. Consequently, Lyme carditis is often overlooked in the differential diagnosis unless patients exhibit high-degree AV nodal conduction abnormalities [4].

In the case we present, a woman reported nonspecific symptoms, including nausea, dizziness, constipation, and fatigue. Work-up for coronary-related etiologies, other infectious etiologies, and inflammatory etiologies was negative. Initial testing revealed a negative ELISA for Lyme disease, but the IgM-specific Western blot for *Borrelia burgdorferi* was positive for 3/3 bands. Among the three *Borrelia*-specific bands, the 39 kDa band is the most specific for *B. burgdorferi* infection and has the strongest correlation with early-stage Lyme disease [3]. Due to the constellation of symptoms, the preceding tick bite, and the negative work-up for alternate etiologies, the diagnosis of Lyme disease was made.

Following discharge, the patient was evaluated in the infectious disease clinic, where repeat testing again confirmed a positive IgM-specific Western blot for *B. burgdorferi*. Given her reported history of tick bites, arthralgias, and intermittent red rashes, she was diagnosed with Lyme carditis. Treatment was initiated with a 21-day course of doxycycline, leading to significant clinical and echocardiographic improvement. 

## Conclusions

This case underscores the critical importance of considering Lyme disease, and specifically Lyme Carditis, in the differential diagnosis of new-onset non-ischemic cardiomyopathy. The patient's significant recovery of left ventricular function following appropriate antimicrobial therapy highlights the potential for reversibility in Lyme carditis-induced cardiomyopathy. Clinicians should maintain a high index of suspicion for Lyme disease in patients presenting with heart failure symptoms, especially when accompanied by a history of tick exposure and relevant clinical manifestations. Early recognition and timely initiation of antibiotic treatment are paramount, as they can lead to substantial cardiac recovery and prevent long-term complications. This case contributes to the growing body of evidence that supports the inclusion of Lyme disease in the evaluation of unexplained cardiomyopathy, advocating for heightened awareness and prompt management to improve patient outcomes. Additionally, patients in endemic areas should be counseled on preventative measures such as wearing long pants in wooded/tall-grass areas, spot-checks for tick bites after being outdoors for a prolonged period of time, and close monitoring of any skin rashes or joint pain. 
